# Epidemiological Characteristics of 69,382 COVID-19 Patients in Oman

**DOI:** 10.1007/s44197-021-00001-9

**Published:** 2021-08-04

**Authors:** Salah T. Al Awaidy, Faryal Khamis, Badria Al Rashidi, Ahmed H. Al Wahaibi, Abdulrahim Albahri, Ozayr Mahomed

**Affiliations:** 1 Office of Health Affairs, Ministry of Health, P.O. Box 393 PC 100, Muscat, Oman; 2 Adult Infectious Diseases, Department of Medicine, Royal Hospital, Ministry of Health, Muscat, Oman; 3 Directorate of Primary Health Care, Ministry of Health, Muscat, Oman; 4 Diwan Health Complex, Muscat, Oman; 5 Department of Public Health Medicine, University of KwaZulu Natal, Durban, South Africa

**Keywords:** COVID-19, Epidemiological characteristics, Pandemic, SARS-CoV-2, Oman

## Abstract

**Objectives:**

To describe the epidemiological characteristics of the first 69,382 patients with COVID-19 infection in Oman.

**Methods:**

A retrospective case series study of patients diagnosed with SARS-CoV-2 infection in Oman from 24 February to 23 July 2020. The data were obtained from the National surveillance COVID-19 network.

**Results:**

The overall mean age of patients was 34 (± 14) years, 74% (*n* = 51,391) were males, 40,859 (59%) were Omani citizens, and 28,523 (41%) were foreign-born individuals. The most common symptoms at presentation were fever 50% (*n* = 34,600), cough 46% (*n* = 32,062), sore throat 46% (*n* = 31,953) and shortness of breath (SOB) 35% (*n* = 24,567). Overall, 8,960 (12.9%) patients required hospitalization with 1189 (13.3%) individuals requiring admission to the intensive care unit (ICU) and mechanical ventilation (MV). Patients hospitalized with COVID-19 infection were mostly Omani nationals and males between 30 and 39 years old (*p* < 0.001). The mortality rate was 7.7 per 100,000 population (*n* = 359) with rates of 9.4 (*n* = 278) and 4.8 (*n* = 81) deaths per 100,000 population in Omani nationals and foreign-born individuals, respectively. Females hospitalized with COVID-19 had a mean age of 64 (± 18) years versus a mean age of 55 (± 17) years in males. Mean age of patients with COVID-19-related mortality was 58 (± 18) years with significant differences in mean age between females and males 64 (± 18) versus 55 (± 17) years, respectively.

**Conclusions:**

Young Omani males accounted for the highest incidence of COVID-19 infection and hospitalization, while mortality rates were higher among males and the older age (> 50 years). Identifying the epidemiological characteristics and outcomes of COVID-19-infected patients is essential for developing targeted intervention strategies and preparing for the resurgence of anticipated second and third waves of this pandemic.

## Introduction

Coronavirus disease 2019 (COVID-19) is a respiratory infectious disease caused by severe acute respiratory syndrome coronavirus 2 (SARS-CoV-2) that has spread worldwide infecting more than 129 million individuals and causing more than 2,778,619 deaths [1].

In Oman, the first two cases were reported on 24 February 2020 from Muscat Governorate (Province), the capital of Oman, and were linked with travel to the Islamic Republic of Iran [1, 2].

As of 24 May 2021, the country has documented 210,364 (4.119/100,000 population) including 194,950 (93%) cases recovered and 2255 deaths with a fatality rate of 1.1% [3].

Oman was among the first countries to implement early and unprecedented precautionary measures to prevent and mitigate the impact of SARS-CoV-2. Despite these interventions, Oman has seen a delayed but exponential increase in COVID-19 cases. In this study, we describe the epidemiological characteristics of the laboratory-confirmed SARS-CoV-2 cases reported between February and July 2020.

## Materials and Methods

### Study Setting

Oman is one of the 22 countries within the Eastern Mediterranean Region (EMR) of the World Health Organization (WHO) that is located in the south-eastern corner of the Arabian Peninsula with a coast that extends 3165 km from the Strait of Hormuz and whose borders include Yemen to the south, and the Kingdom of Saudi Arabia and United Arab Emirates to the west. The country has a population of nearly 5 million out of which 39% are foreign-born individuals (Expatriates). Out of the total, 2,739,954 (61.3%) are males, and 1,731,194 (38.7%) are females. While children under 10 years of age represent around 19% of total Omani population, 13% of the population is between age 10 and 15 years, 65% between age 15 and 50 years and 3% are above 50 years [4].

### Case Inclusion Definition

In this retrospective case series study, we reviewed data of 69,382 laboratory-confirmed COVID-19 cases between 24 February and 23 July 2020. The surveillance data were notified by all healthcare institutions to the National Department of Communicable Diseases, where the data are compiled, analysed and disseminated at national level [5].

The diagnosis of COVID-19 infection was based on the National Case Definitions for Suspected and Confirmed COVID-19 Interim Guideline [5]. In addition, all cases included during this study period were laboratory-confirmed by SARS-CoV-2 real-time reverse transcriptase-polymerase chain reaction (RT-PCR).

### Data Collection

Data were retrieved from published national surveillance data during the study period [5]. A Microsoft excel template was used to extract the variables regarding demographic characteristics (gender, age, place of residency and nationality), clinical presentation and patients’ outcomes (hospitalization, mortality and recovery). We also reviewed the number of cases reported per day, and the number of SARS-CoV-2 PCR tests performed. The principal investigator reviewed the data for completeness, accuracy and missing information. The data were then exported to STATA 13 for statistical analysis.

### Statistical Analysis

Patient characteristics were described using frequency and percentage for categorical variables, mean and standard deviation for continuous variables. Associations between categorical variables were assessed either by using Chi-square test or Fisher's exact test, whereas continuous variables were analysed by the Student's t test or a priori two-tailed level of significance was set at 0.05. Statistical analyses were conducted using STATA version 13.1.

### Ethical Approval

We used anonymized data, and thus, the ethical study approval was not required as it was based on secondary data extracted from the national surveillance system published in an official domain**.** This study does not contravene the internal institutional review board and adheres to the Declaration of Helsinki.

## Results

### Incidence of COVID-19 Cases in Oman

By 23 July 2020, there were 69,382 laboratory-confirmed cases of COVID-19 reported (1502 cases per 100,000 population). Of these, 40,859 (59%) were Omani nationals, and 28,523 (41%) were foreign-born (non-citizen) individuals. Out of the total confirmed cases, 8960 (12.9%) were hospitalized (Table 1).

**Table 1:** Demographic characteristics of Oman’s COVID-19 patients

Characteristic, *n* (%)	Full cases series (*N* = 69,382) (1.5^a^)	Non-hospitalized (*n* = 60,422) (1.3^a^)	Hospitalized (*n* = 8960) (194^a^)	*p*-value
Demographics				
*Age*				
0–9 years	3170 (5%)	1994 (63%)	1176 (37%)	
10–19	3056 (4%)	2831 (93%)	225 (7%)	
20–29	15,678 (23%)	14,949 (95%)	729 (5%)	
30–39	29,102 (42%)	27,606 (95%)	1496 (5%)	
40–49	11,017 (16%)	9591 (87%)	1426 (13%)	
50 +	7359 (11%)	3451 (47%)	3908 (53%)	
*Gender*				
Female	17,991 (26%)	14,992 (83%)	2999 (17%)	< 0.001
Male	51,391 (74%)	45,430 (88%)	5961 (12%)	
*Nationality*				
Omani	40,859 (59%)	34,250 (84%)	6609 (16%)	< 0.001
Non-Omani^b^	28,523 (41%)	26,172 (92%)	2351 (8%)	
*Outcome*				
Died	359 (0.5%)	–	–	
Recovered	47,909 (69.1%)			
Active cases	21,114 (30.4%)			
*Governorate*				
Muscat	36,366 (52.4%)(2558^a^)	–	–	
North Al Batinah	10,968 (15.8%)(1402^a^)	–	–	
South Al Batinah	7844 (11.3%)(1792^a^)	–	–	
Al Dakhiliyah	4063 (5.9%)(837^a^)	–	–	
Dhofar	2608 (3.8%)(577^a^)	–	–	
South Ash Sharqiyah	2074 (3%)(647^a^)	–	–	
North Ash Sharqiyah	1767 (2.5%)(622^a^)	–	–	
Al Wusta	1556 (2.2%)(3151^a^)	–	–	
Adh Dhahirah	1321 (2%)(591^a^)	–	–	
Al Buraimi	769 (1.1%)(655^a^)	–	–	
Musandam	46 (0.1%)(101^a^)	–	–	

*SD* standard deviation

^a^Rate/100,000

^b^Non-Omani included 22.1% (*n* = 15,361) from Indian, 12.8% (*n* = 8905) from Bangladeshi, 2.8% (*n* = 1919) from Pakistani, 0.8% (*n* = 561) from Nepalees, 0.6% (*n* = 429) from Egyptian, 0.5% (*n* = 355) from Filipino, 0.3% (*n* = 212) from Sri Lankan, 0.1% (*n* = 77) from Syrian, 0.1% (*n* = 72) from Tanzanian, 0.9% (*n* = 632) from others

Between 4 March and 15 July 2020, the overall COVID-19 notification rate for the new cases increased significantly from 4 to 846 per 100,000 population (*p* < 0.001). During the same period, the trend of the notification rate of COVID-19 cases increased among Omani nationals. There were a 929-fold increase (from 7 to 929 cases per 100,000 population) for foreign-born individuals and a 1000-fold increase (from 0.4 to 403 increase by 40,260 cases per 100,000 population) for Omani nationals.

**Fig. 1:**
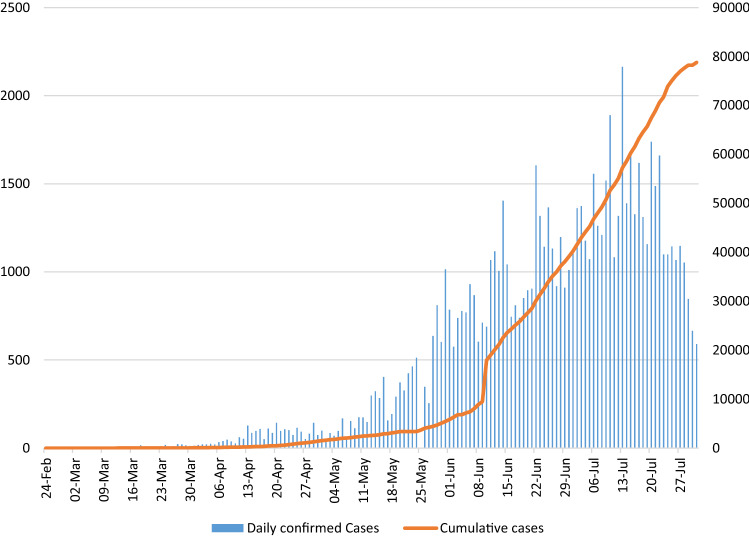
Laboratory-confirmed COVID-19 cases in Oman from 23 February to 30 July 2020

There has been a significant increase in the average number of new cases reported per day with a peak of 1260 cases per day in July 2020. In June, there were 19,453 more new cases detected than May, with an average of 3.25 times more cases reported per day in June than May 2020. Similarly, the cumulative number of SARS-CoV-2 PCR tests performed was 321,051 with an average of 54,000 tests per day (Fig. 1).

**Fig. 2:**
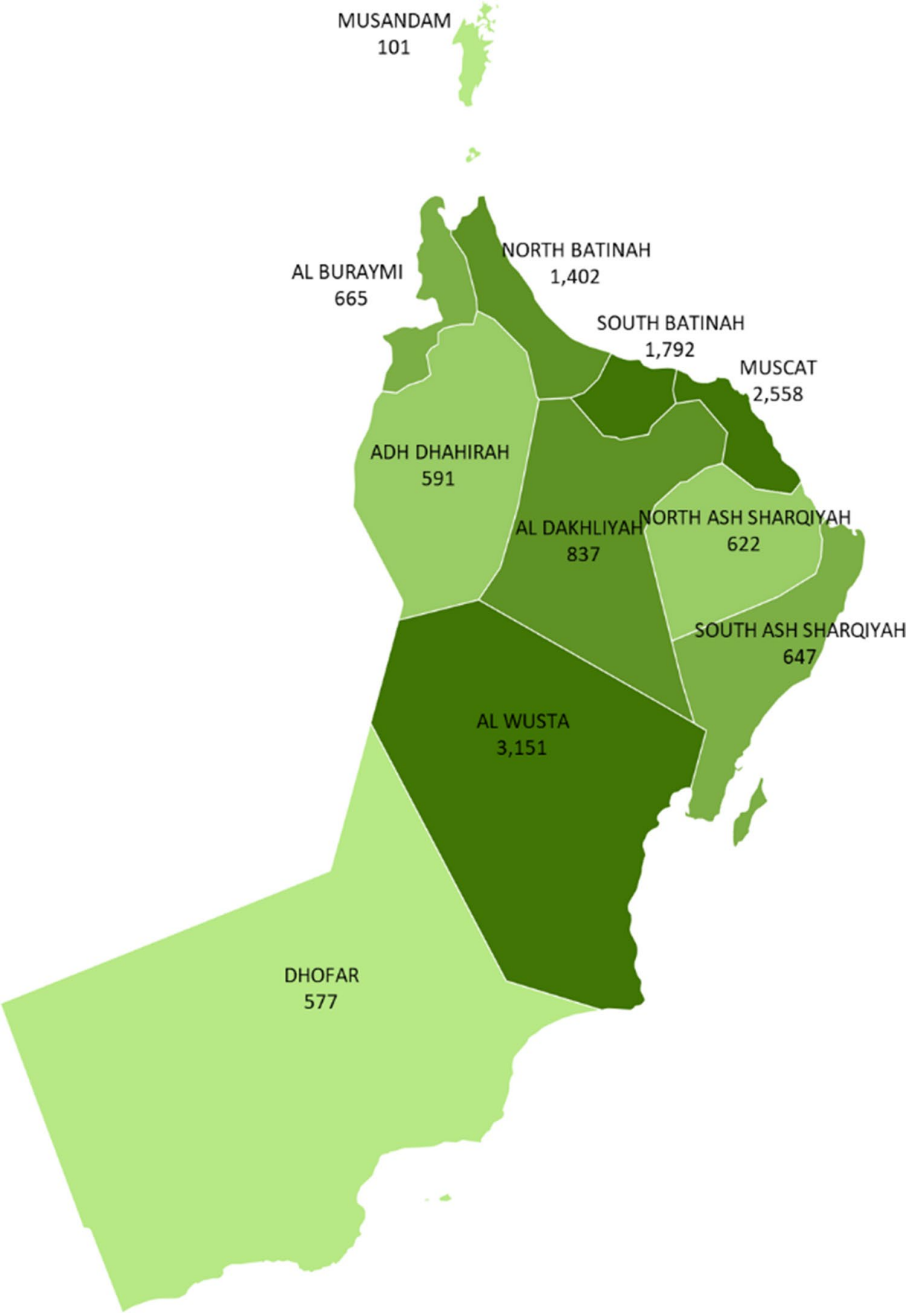
Rates of COVID-19 per 100,000 by governorates (provinces)

There was a substantial variation in COVID-19 rates across the country, with some governorates reporting an incidence as high as 2558 per 100,000 in Muscat to as low as 101 per 100,000 population in Musandam (Fig. 2). Oman reported an average of four new cases of COVID-19 per day in March, which increased to 846 cases per day by July 2020.

#### Demographic and Clinical Characteristics of All COVID-19 Cases

The overall mean (SD) age of the patients was 34 (± 14) years. Only 5% (*n* = 3581) of cases were reported among children below 10 years of age, and an additional 5% (*n* = 3590) of cases were reported among children aged 10–18 years of age (Table 1).

**Fig. 3:**
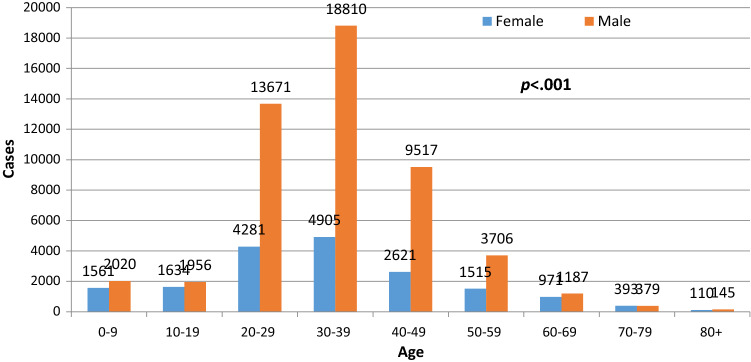
Frequency distribution of COVID-19 cases by age categories and gender

There was a significant difference in the overall mean age of the non-hospitalized [35.2 (± 15.8)] and hospitalized patients [44.4 (± 23.8)] years (*p* < 0.01) (Table 1). About 74% (*n* = 51,931) of the patients with confirmed COVID-19 were males, with a majority (75%) of the confirmed cases between the ages of 20 and 49. Of the total patients with COVID-19, 34% were between the ages of 30 and 39. There were significantly more males diagnosed with COVID-19 than females between age 30 to 39 years old (*p* < 0.001) (Fig. 3).

**Fig. 4:**
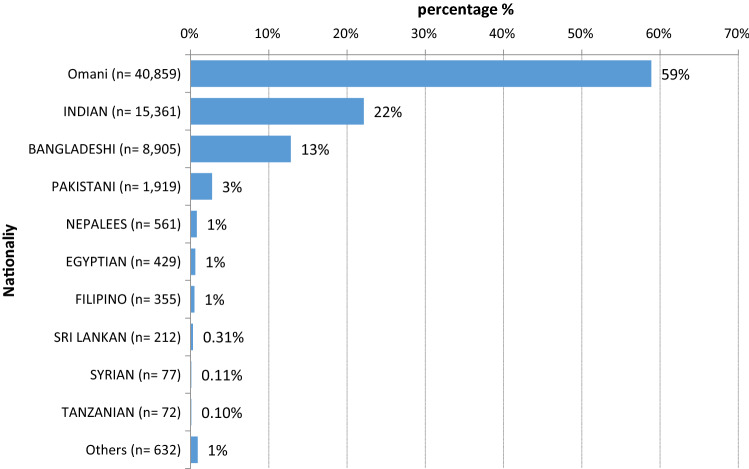
Distribution of COVID-19 cases by nationality

Among the 28,523 foreign-born individuals, Indians represented 22% (*n* = 15,361), Bangladeshi 12% (*n* = 8905), Pakistani 2% (*n* = 1191) with 9% from other nationalities (Fig. 4).

**Fig. 5:**
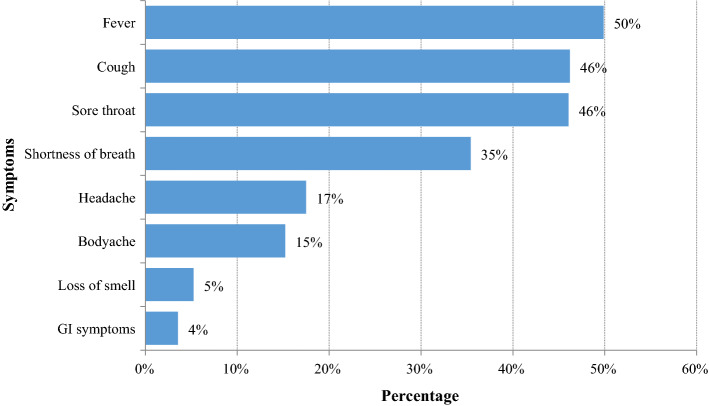
Symptoms of COVID-19 patients on diagnosis (*N* = 69,382)

The most common reported symptoms on notification were fever 50% (*n* = 34,600), cough 46% (*n* = 32,062), sore throat 46% (*n* = 31,953) and shortness of breath 35% (*n* = 24,567) (Fig. 5). A majority 87% of (*n* = 60,422) patients were treated as outpatients, and 8950 (13%) were hospitalized.

At the end of July 2020, 69% (*n* = 47,909) of patients had completely recovered, 0.5% (*n* = 359) had died, while 30.5% (*n* = 21,114) were still active cases (Table 2). The mean age of patients who recovered was 34 (± 14) years, with no significant difference between the mean age of males 34 (± 12) and females 33 (± 17) years. The recovery rate was 83% (23,914/28,523) among foreign-born patients and 59% (23,995/40,859) among Omani nationals (*p* < 0.001) (Table 2).

**Table 2:** Mortality of all COVID-19 cases, Oman

Characteristic, *n* (%)	Recovered		*p*-value	Dead		*p*-value
	Male	Female		Male	Female	
Gender	37,007	10,902		278	81	
Age mean (SD)	34 (13)	33 (17)	1.00	55 (17)	64 (18)	< 0.001*
*Nationality*						
Omani Nationals	14,597	9398	< 0.001	133	73	< 0.001
Non-Omani	22,410	1504		145	8	
*Governorate*						
Muscat	23,189	5698		151	34	
North Al Batinah	3710	1719		49	20	
South Al Batinah	3065	1641		42	12	
AL Dakhiliyah	1695	731		10	4	
Dhofar	1247	64		6	2	
South Al Sharqiyah	1049	383		9	7	
North Ash Sharqiyah	633	330		2	0	
AL Wusta	1379	9		1	0	
Adh Dhahirah	596	202		1	1	
AL Buraimi	413	123		7	1	
Musandam	31	2		0	0	

### Demographics and Clinical Characteristics of Patients Hospitalized with COVID-19

Out of the total number of diagnosed patients, 8960 (194 cases per 100,000 population) required hospitalization, among whom 1189 (13.3%) required admission to the ICU and subsequently required mechanical ventilation (MV) (Table 3). The ICU admission rate was 1.71% of total COVID-19-infected patients. Of the patients requiring MV, 88% (*n* = 992) were between 30 and 79 years of age, and only 4.8% (*n* = 43) were under 19 years old.

**Table 3:** Demographic characteristics of hospitalized COVID-19 patients in Oman. Stratified by intensive care unit (ICU) admission

Characteristic, *n* (%)	All (*N* = 8960)(194^a^)	Non-ICU (*n* = 7771)	ICU (*n* = 1189)	*p*-value
Demographics				
*Age*				
Mean (SD)	44.4 (23.8)	43.3 (24.3)	51.7 (19.2)	
< 20 years	1401 (16%)	1336 (95%)	65 (5%)	< 0.001
20–39 years	2225 (25%)	1990 (89%)	235 (11%)	
40 years and above	5334 (60%)	4445 (83%)	889 (17%)	
*Gender*				
Female	2999 (33%)	2689 (90%)	310 (10%)	< 0.001
Male	5961 (67%)	5082 (85%)	879 (15%)	
*Nationality*				
Omani	6609 (74%)	5846 (88%)	763 (12%)	< 0.001
Non-Omani^b^	2351 (26%)	1925 (82%)	426 (18%)	
*Hospital location*				
Muscat	2960 (33%)	2535 (85.6%)	425 (14.4%)	–
North Al Batinah	1311 (14.6%)	1159 (88.4%)	152 (11.6%)	
Dhofar	1175 (13.1%)	1016 (86.5%)	159 (13.5%)	
South Al Batinah	968 (10.8%)	850 (87.8%)	118 (12.2%)	
Al Dakhiliyah	849 (9.5%)	609 (71.7%)	240 (28.3%)	
South Ash Sharqiyah	548 (6.1%)	496 (90.5%)	52 (9.5%)	
North Ash Sharqiyah	385 (4.3%)	368 (95.6%)	17 (4.4%)	
Adh Dhahirah	291 (3.2%)	277 (95.2%)	14 (4.8%)	
Al Buraimi	234 (2.6%)	222 (94.9%)	12 (5.1%)	
Al Wusta	185 (2.1%)	185 (100.0%)	0 (0.0%)	
Musandam	54 (0.6%)	54 (100.0%)	0 (0.0%)	
*Length of stay*				
Average (SD)	2.1 (2.9)	1.9 (2.6)	3.1 (4.2)	
0–3 days	7430 (83%)	6570 (88%)	860 (12%)	< 0.001
4–7 days	1092 (12%)	901 (83%)	191 (17%)	
8+ days	438 (5%)	300 (68%)	138 (32%)	

*SD* standard deviation

^a^Rate/100,000

^b^Non-Omani included 11.4% (*n* = 1017) from Bangladeshi, 7.9% (*n* = 706) from Indian, 2.9% (*n* = 259) from Pakistani, 0.9% (*n* = 82) from Yemeni, 0.9% (*n* = 81) from Egyptian, 0.4% (*n* = 36) from Filipino, 0.2% (*n* = 18) from Syrian, 0.2% (*n* = 16) from Sudanese, 0.2% (*n* = 15) from Nepalees, 0.1% (*n* = 13) from Tanzanian, 1.2% (*n* = 108) from others

The overall mean age of hospitalized patients was 44.4 (± 23.8) years of age, while those admitted to ICU were slightly older (52 ± 19.2 years of age) (Table 3). Males (67%; *n* = 5961) comprised the largest proportion of admissions, with the majority of admitted patients (75%; *n* = 6609) being Omani nationals.

During the same period, the cumulative COVID-19 hospitalization rate for children < 19 years of age was 29 per 100,000 population (*n* = 1336) compared to 165 per 100,000 population (*n* = 7624) for adults aged 19 years and above. About 83% (*n* = 7430) were admitted for < 3 days (*p* < 0.001) (Table 3).

There were substantial variations in hospitalization rates across the country, with some governorates reporting an admission rate as high as 260 to as low as 119 cases per 100,000 population in Dhofar and Musandam Governorates, respectively (Table 3). Muscat (33%; *n* = 2960), North Al Batinah (15%; *n* = 1311) and Dhofar (13.1%; *n* = 1175) were the three leading governorates with respect to hospitalization of COVID-19 patients.

### COVID-19-Related Mortality

The mortality rate up to 23 July was 7.7 per 100,000 population (*n* = 359). The COVID-19 mortality rates were 9.4 (*n* = 278) and 4.8 (*n* = 81) deaths per 100,000 population in Omani nationals and foreign-born individuals, respectively. The death rate increased by 15%, in March representing a rate of 0.02 deaths per 100,000 population, and this increased to 0.2 deaths per 100,000 population by the end of July. However, the death notification rate remained below 2 cases per million population from March to July 2020.

The mean age of patients with COVID-19-related mortality was 58 (± 18) years, with significant differences in the mean age between females and males, 64 (± 18) versus 55 (± 17) years, respectively (*p* < 0.001). Mortality from COVID-19 was higher among Omani nationals 58% (*n* = 206), with the Governorate of Muscat accounting for 51% (*n* = 185) of the total deaths (Table 2).

## Discussion

We describe the epidemiological characteristics of the initial 69,382 laboratory-confirmed COVID-19 patients in Oman and provide additional insight into the epidemiological presentation and outcomes of COVID-19 patients compared to an earlier study [2]. Khamis and colleagues in a retrospective case series that were conducted over a 6-month period reported an incidence rate of laboratory-confirmed COVID 19 of 1502 cases per 100,000 population. The rate was lower than other Gulf countries such as Bahrain, Qatar and Saudi Arabia that documented rates of 2387, 3942 and 7893 cases per 100,000 population, respectively. The rates in Oman were similar to Kuwait (1561 per 100,000 population) and higher than UAE (607 cases per 100,000 population) [6]. Internationally, at that time period countries that reported lower rates included Canada (286 cases per 100,000) [7], Taiwan (1.9 cases per 100,000) and the USA (1014 cases per 100,000) [8].

**Fig. 6:**
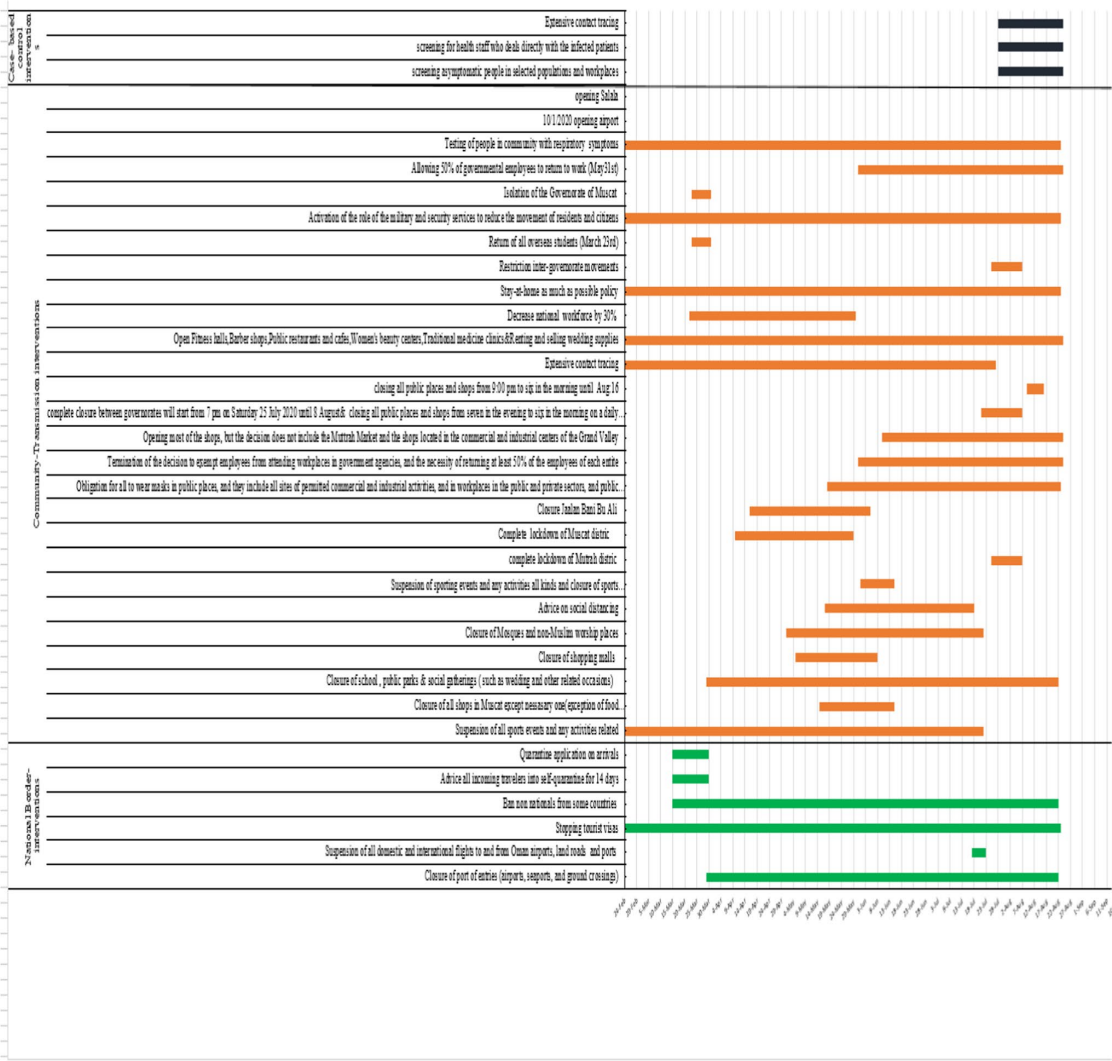
Non-pharmaceutical interventions implemented along with the dates of various strategies to minimize its spread in Oman

Following detection of the first case on 24 February 2020, the country implemented a number of unprecedented non-pharmaceutical interventions (NPIs) (Fig. 6] such as case-based control interventions that included early case detection, isolation of suspected and confirmed patients, contact tracing and enhancing laboratory diagnostics. National border interventions were commenced on 12 March 2020. The country implemented travel restrictions, initially to countries with a high burden of disease such as China followed by suspension of all international flights, entry bans through borders and ports, stopping tourist visas and advising all incoming travellers to self-quarantine for 14 days. Community transmission interventions that included intergovernmental travel restrictions were deployed across the country on April 1 2020, enforcing infection control interventions across the nation such as universal masking, maintaining social distancing and reducing non-essential national workforce by 30%, closure of amusements, schools, malls, mosques, restrictions on social gatherings and postponement of large public events and mass gatherings such as weddings, conferences and governmental events.

However, despite governmental implementation of these interventions, the reported cases continued to rise. A number of factors could be the reason behind increased rates of confirmed COVID-19 cases in Oman, including the enhancement of the national COVID-19 surveillance system and the extensive availability of diagnostic testing for early detection of cases could be the reasons; lack of adherence among the public to NPI such as social distancing, gathering and universal masking, particularly among family members and HCWs or delay in mitigation interventions among vulnerable populations are other important potential factors. In addition, a recent viral genomic study from Oman indicated the predominance of the spike D614G variant that has been associated with increased transmission [9]. Gender, a biological attribute and social construct, may also influence an individual’s susceptibility, vulnerability and exposure to infectious diseases [10–12].

In this study, COVID-19 was more predominant among young adult males with a mean age of 34 (± 14) years. This has been similar to the average age of 36 years that was reported in several studies [13–16]. The predominance of COVID 19 in males and younger age groups is reflective of the population distribution in Oman where the majority of population are young [17]. In addition, young males constitute a large part of the working force in the country and tend to be mobile and socially interactive with others.

Similar to other reports [13, 14, 18], the three most common COVID-19 symptoms on initial presentation were fever (76%), cough (48%) and sore throat (46%) reflecting the mild nature of the illness in most of our patients.

While the initial reports from Oman indicated a predominance of COVID-19 in foreign-born individuals, this study showed that majority (59%) of the confirmed COVID-19 cases were among Omani nationals. The possible explanation for the lower proportion of cases among the foreign-born population (expatriates) could be the demographic make-up, with most expatriates being younger in age. Additionally, expatriates may be less inclined to go for a test or to hospitals due to fear of victimization or loss of employment or due to lack of health insurance or inadequate information and other cultural barriers [19]. Furthermore, the NPI measures adopted by the country, which included lockdown of areas heavily populated with foreign-born individuals, probably resulted in the decrease in infection rates. However, similar measures among nationals were ineffective, which may be attributed to the frequent social gatherings among extended family members despite repeated advices from the health authorities.

In comparison with all other governates, Muscat Governorate reported the highest rates of COVID-19. The governorate has unique demographic, socioeconomic and community features as it has the largest population with the highest density and many foreign-born individuals, mostly single labourer men living in very crowded houses with poor living conditions [2].

Our study supported the finding that men are more likely to become infected by COVID-19, potentially due to sex-based immunological differences [20], gender variations or associated comorbidities including hypertension, cardiovascular disease, lung disease, patterns and prevalence of smoking [21], drinking alcohol and other behaviours commonly associated with masculine norms [22]. Similar to earlier studies, patients hospitalized with COVID-19 infection were significantly more likely to be males [3, 13, 23–25] and of the age group between 30 and 39 years old (*p* < 0.001) [26].

In this case series, 13% of the patients required hospitalization with 13.3% of our hospitalized patients required ICU admission and MV; compared with 4.7% in Saudi Arabia [13], 8.7% in California [27], 14% in New York City area, 24% across the USA and up to 20% in China [28–31]. Although our hospitalization rates were low, higher numbers of COVID-19 patients needed ICU care [16]. This could be due to high rates of associated comorbidities such as diabetes mellitus, hypertension and chronic kidney disease in our population [32]. Additionally, a delay in presentation and admission to the hospitals could have been attributable factors particularly as the country adopted home quarantine early on and throughout the pandemic.

Similar to other global studies, 49% of our patients who required MV were above the age of 59. A review conducted among confirmed COVID-19 patients admitted to a hospital in Germany between 26 February and 19 April 2020 showed that 24% of patients between 60 and 69 years of age, 25% of patients between age 70 and 79 years and 12% of patients of age 80 years and above required ICU admission [33]. In another study from the USA, 27.4% of patients from the age group of 60–69 age group were admitted to the ICU [16].

During the study period, Oman experienced a sharp rise in COVID-19-associated deaths (78 per 1,000,000 population). The number of excess deaths reported could reflect an increase in rates of death directly caused by the disease. The mortality rates of COVID-19 were higher than China (3.3/1,000,000) [34], the EU/EEA and the UK (4.1 per 1,000,000 population; country range 0–15.9) [36]. In comparison with Oman, Bahrain, Kuwait, Saudi Arabia and Iran have documented much higher mortality rates of 85, 105, 819 and 199 cases per 1,000,000 population, respectively. On the other hand, Qatar and UAE have reported lower mortality rates than Oman reaching 35 and 61 cases per 1,000,000 population, respectively [6]. The differences in death rates possibly attributed to the timely access to the health facilities in different countries and the resilience of the healthcare systems. In Oman, the death rate has increased over the study period by 150% from 0.2 deaths per 1,000,000 population in March to 1.7 deaths per 1,000,000 population in the end of July 2020. Early diagnosis and case detection, timely care and risk stratification of patients who may progress to severe illness are important factors to reduce the mortality rates.

In this study, we noted significant gender difference in the risk of acquisition of COVID-19 infection among males and females. For reasons that remain unclear, the novel coronavirus appears to present a significantly higher mortality risk in men than in women [13, 23–25, 33, 35]. Similar to other international studies, our findings indicate that 69% of all admission to hospitals were males accounting for 74% of all ICU admissions and 77% of all mortality for COVID 19 in Oman. Research in many countries prior to COVID 19 indicated that women have more healthcare utilization than men [36, 37].

In our study, the mean age of patients with COVID-19-related mortality was 58 (± 18) years, with females mean age of 64 (± 18) years and males mean age of 55 (± 17) years. This is similar to findings from a longitudinal cohort from the USA that included 88,747 COVID-19 cases confirmed by PCR between 28 February and 14 May 2020, where patients older than 50 years had a progressively higher mortality compared to patients less than 50 years [38].

To date, this study is the largest study to report the clinical and epidemiological characteristics of laboratory-confirmed COVID-19 patients from Eastern Mediterranean Region (EMR) region. However, the findings are subject to a few limitations. First, because this is a retrospective study, there may be biases related to patient recall, transcription error or missing information that were introduced during a patient encounter. Second, some data on symptoms, comorbidities and risk factors were missing in records from both non-hospitalized and hospitalized patients. Third, the impact of an infection depends not only on the number of individuals infected but also the infection’s transmissibility and the spectrum of clinical severity, and these variables were not collected in this study. Fourth, information on the treatment given to hospitalized patients was not available, which could affect the patient outcomes. Finally, hospitals in the governates had different admission criteria, which limited the number of patients enrolled.

## Conclusions

To our best of knowledge, this is the largest retrospective case series study describing the epidemiological characteristics of COVID-19 patients in the EMR region. The findings of this study in terms of demographic profile, hospitalization and mortality rates are in keeping with data published from other countries. We found that patients hospitalized with COVID-19 were significantly more likely to be Omani citizens, male and between 30 and 39 years of age. These findings underscore the importance of public health interventions that prevent transmission, mitigate hospital surges and reduce the impact of the second and subsequent waves of COVID-19 infection in this pandemic.

## Data Availability

The data that support the findings of this study are available from the corresponding author (Salah T. Al Awaidy), upon reasonable request.
